# Modelling of strategies for the introduction and routine use of multivalent meningococcal conjugate vaccines (MMCVs) in the African meningitis belt

**DOI:** 10.1371/journal.pone.0330627

**Published:** 2025-08-29

**Authors:** Andromachi Karachaliou Prasinou, Caroline Trotter

**Affiliations:** Disease Dynamics Unit, University of Cambridge, Cambridge, United Kingdom; Menzies School of Health Research: Charles Darwin University, AUSTRALIA

## Abstract

The introduction of MenAfriVac has significantly reduced group A meningococcal meningitis in the African meningitis belt, but epidemics caused by other groups such as C, W, Y and X (MenCWYX) remain a threat. To address this, a new multivalent meningococcal conjugate vaccine (MMCV) has been developed and pre-qualified by WHO. This study extends a previously established transmission dynamic model for MenA to include MenCWYX, enabling evaluation of the potential impact of MMCVs under various vaccination strategies. Using Burkina Faso as a case study, the model simulates mass campaigns targeting different age groups and routine vaccination through the Essential Programme on Immunization (EPI). The results indicate that campaigns targeting 1–29-year-olds are most effective in averting cases and delaying disease resurgence, while 1–19-year-old campaigns offer a resource-efficient alternative. Vaccine efficacy against carriage and the duration of protection significantly influence outcomes; greater efficacy (90% vs. 60%) and longer protection delay resurgence and reduce the number of cases. Routine- only vaccination demonstrates value in lower-risk settings, though it is less effective than combined strategies. Sensitivity analyses confirm the robustness of the ranking of strategies but highlight the importance of accurate estimates of vaccine efficacy and transmission parameters. The findings suggest that countries in the meningitis belt should integrate MMCVs into their immunisation programs, with high-risk countries prioritising catch-up campaigns for children and young adults. Despite data limitations and uncertainties, this model provides valuable insights for optimising vaccine rollout and highlights critical research needs, such as understanding vaccine effectiveness against carriage. These results support informed decision-making to sustain progress against meningitis and protect populations from future epidemics. MMCVs hold great promise in further reducing meningitis burden and approaching disease elimination in the region.

## Introduction

The widespread introduction and routine use of MenAfriVac, a monovalent group A meningococcal conjugate vaccine, has been a great success in reducing the burden of epidemic meningitis in the African meningitis belt [1,2]. However, protection against group A needs to be maintained and there remains a threat from other meningococcal serogroups [3], with large outbreaks of group C and group W in recent years. To counter this threat, a new, affordable multivalent meningococcal conjugate vaccine [4] was developed by the Serum Institute of India; this was pre-qualified by WHO in July 2023 [5]. In 2018, the Gavi Board conditionally approved support for the use of MMCVs in a targeted approach [6]. Now that a vaccine is available, evidence-based recommendations are required to guide appropriate use of MMCVs in the meningitis belt.

Transmission dynamic models of group A meningitis (menA) were previously developed to inform long-term immunisation strategies with MenAfriVac, following introductory campaigns in 1–29 year olds [7]. These illustrated that ongoing vaccination efforts, e.g., through the Essential Programme on Immunization (EPI) are critical. Furthermore, this model highlighted the importance of considering indirect (herd) protection arising from reductions in meningococcal carriage as well as direct protection in immunised individuals. There was excellent evidence of MenAfriVac’s impact on carriage from the MenAfriCar Consortium’s study, however, the impact of the new MMCV on carriage has not yet been evaluated. Based on the experience with MenAfriVac in Chad [1] and quadrivalent meningococcal conjugate vaccines in the UK [8], vaccine effectiveness against carriage could be expected to be between 60% and 90%.

Here, we adapted the group A model to include the transmission dynamics of other groups (CWYX) to evaluate the potential impact of MMCVs in the meningitis belt, accounting for the earlier use of MenAfriVac. This paper particularly aims to investigate the impact of targeting different age groups for MMCVs introductory campaigns.

## Methods model structure

The constructed model is based on a system of ordinary differential equations. Similarly to our previous work [7], the model divides the population into mutually exclusive subpopulations according to their infection status.

**Fig 1:**
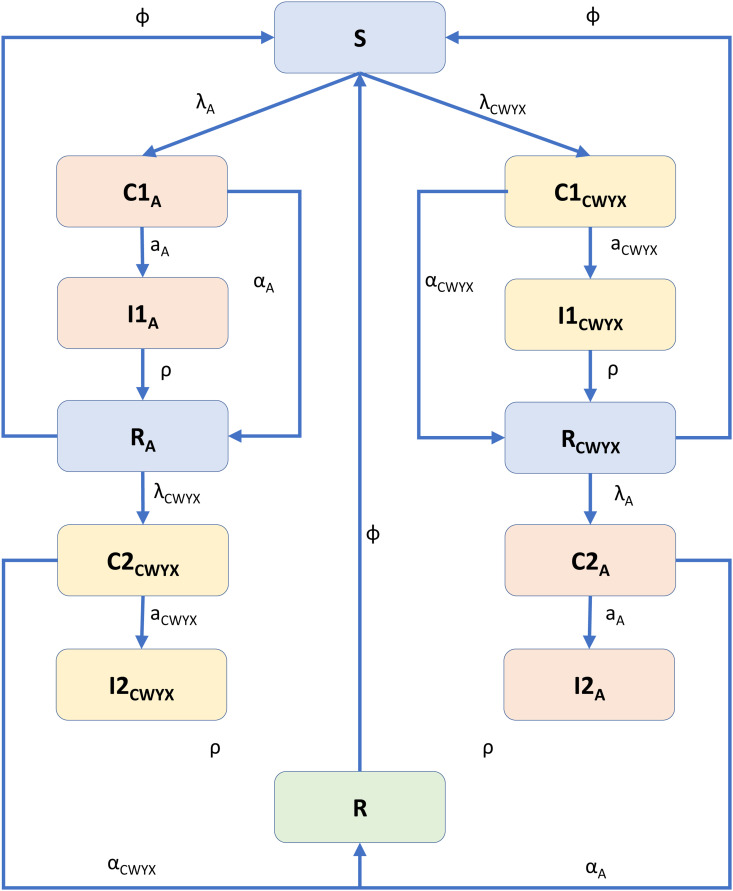
Diagram of the model for *Neisseria meningitidis* transmission and disease. Each compartment is divided into distinct age classes (not shown here).

In the adapted model, we have combined groups C, W, Y and X together (menCWYX). The model structure for menA is replicated so that there are additional compartments for menCWYX susceptibles, carriers, invasive disease and recovered/ immune (Fig 1). Following contact with an infected person, susceptibles (S ) may become a carrier of either menA ($C1_{A}$) or menCWYX ($C1_{CWYX}$). We assume that there is no carriage of two groups at the same time. An episode of carriage leads to a state with temporary immunity from future re-infections with the same serogroup ($R_{A},\;R_{CWYX}$), while occasionally it may lead to invasive disease ($I1_{A}\text{,\textbackslash{} }I1_{CWYX}$). People with temporary natural immunity against one group are assumed to be susceptible against the other group and may further become carriers and/or develop invasive disease ($C2_{A}$, $C2_{CWYX}$, $I2_{A}$, $I2_{CWYX}$). A second infection with a new group leads to a state with natural immunity against all carriage and disease (R ). All the states and their description can be seen in Table 1 and the description of the model parameters are shown in Table 2. The population is further structured by age into 100 annual age cohorts.

**Table 1:** Description of the model states.

State	Description
S	Susceptible
C1_A_, C2_A_	Carrier with group A
I1_A_, I2_A_	Ill with group A
R_A_	Recovered from to group A
C1_CWYX_, C2_CWYX_	Carrier with group C, W, Y or X
I1CWYX, I2_CWYX_	Ill with group C, W, Y, or X
R_CWYX_	Recovered from groups C, W, Y and X
R	Recovered from all serogroups

**Table 2:** Description of the model parameters.

Parameter	Value	Description
$\lambda_{A}$	Age-specific	Force of infection for group A
$\lambda_{CWYX}$	Age-specific	Force of infection for groups C, W, Y and X
a_A_	Age-specific	Rate at which carriers fall ill. Specific for group A
a_CWYX_	Age-specific	Rate at which carriers fall ill. Specific for groups C, W, Y and X
ρ	52/year	Recovery rate from disease
ϕ	0.0839/year	Rate of loss of natural immunity
$\alpha_{A}$	12/year	Rate of loss of carriage. Specific for group A
$\alpha_{CWYX}$	6/year	Rate of loss of carriage. Specific for groups C, W, Y and X

**Fig 2:**
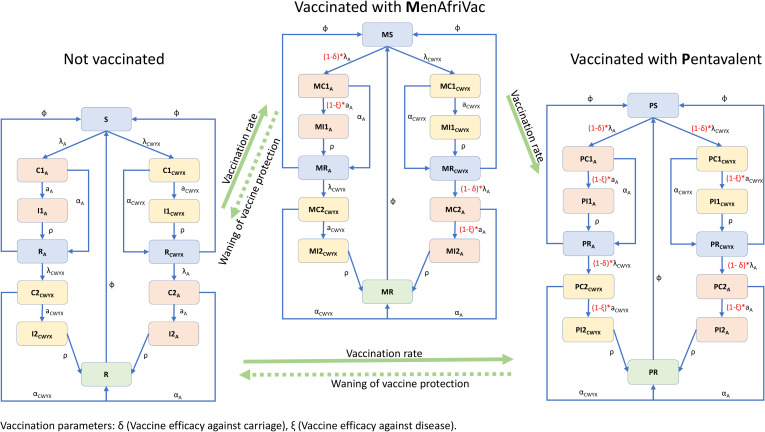
Diagram showing the implementation of the vaccination strategy.

On introduction of vaccines, the population is further divided into vaccinated states which mirror the unvaccinated states (Fig 2). Vaccinated states with MenAfriVac have the prefix M while vaccinated states with MMCV have the prefix P. People move from the unvaccinated states to the vaccinated ones upon receiving a vaccine dose. Vaccinated individuals are not perfectly protected but their risk of infection is lower relative to unvaccinated individuals. Individuals initially receive MenAfriVac, which results in lower risks of acquisition and disease with menA only. This is followed by vaccination with the novel MMCV which offers protection against all 5 groups. Vaccine protection wanes over time and people revert back to the unvaccinated states. The full system of ordinary differential equations can be found in the supplementary material.

### Parameters

Based on empirical data, we assume that the duration of carriage with serogroups C, W, Y or X is 2 months, compared to 1 month duration assumed for menA [9].

The age-specific force of infection for menA in this model is given by:


$$\lambda_{j_{A}}=\theta \sum_{k=1}^{n}\beta_{A}(z_{j},z_{k})({C1}_{A_{k}}+{I1}_{A_{k}}+{C2}_{A_{k}}+{I2}_{A_{k}}+{MC1}_{A_{k}}+{MI1}_{A_{k}}+{MC2}_{A_{k}}+{MI2}_{A_{k}}+{PC1}_{A_{k}}+{PI1}_{A_{k}}+{PC2}_{A_{k}}+{PI2}_{A_{k}})$$

Where $\beta_{A}(z_{j},z_{k})$ is the contact rate between people in the $j^{th}$ and $k^{th}$ age classes and θ is the stochastic term as described in our previous model [7]. The values of the WAIFW social contact matrix as well as the rate at which carriers with menA develop invasive disease have remained the same as in our original model. For consistency, we recalibrated the value of the transmission parameter $\beta_{A}$ so the average disease burden is the same as the previous menA-only model.

The age-specific force of infection for menCWYX is given by:

$\lambda_{j_{CWYX}}=\theta \sum_{k=1}^{n}\beta_{CWYX}(z_{j},z_{k})({C1}_{{CWYX}_{k}}+{I1}_{{CWYX}_{k}}+{C2}_{{CWYX}_{k}}+{I2}_{{CWYX}_{k}}+{MC1}_{{CWYX}_{k}}+{MI1}_{{CWYX}_{k}}+{MC2}_{{CWYX}_{k}}+{MI2}_{{CWYX}_{k}}+{PC1}_{{CWYX}_{k}}+{PI1}_{{CWYX}_{k}}+{PC2}_{{CWYX}_{k}}+{PI2}_{{CWYX}_{k}})$.

Data suggest that in the absence of any vaccination, 80% of the total number of cases predicted by the model are due to menA while the remaining 20% are due to groups C, W, Y or X [10]. Since the duration of carriage for groups C, W, Y and X is assumed to be twice as long as for group A, to maintain this relative disease burden, we recalibrated the transmission parameters $\beta_{CWYX}$ and $a_{CWYX}$. This translates into 40% transmission rate and 20% invasion rate relative to group A. We examined this in a sensitivity analysis which considered 60% transmission rate and 10% invasion rate relative to group A.

Due to lack of any evidence suggesting otherwise, the duration of invasive disease as well as the duration of natural immunity were assumed to be the same for all groups.

### Vaccine effectiveness

Vaccination with either MenAfriVac or MMCV offers some level of protection against infection. In vaccinated populations with MenAfriVac, the transmission rate $\beta_{A}$ is reduced by a factor of 1-$\delta_{A}$ and the rate at which carriers develop invasive disease $a_{A}$is reduced by a factor of 1-ξ. We assumed 90% vaccine efficacy against carriage and disease with MenAfriVac [1]. In the base case, we assumed 90% vaccine efficacy for MMCV as well and changed that to 60% vaccine efficacy against carriage ${(\delta }_{CWYX})$ in a sensitivity analysis.

### Model implementation

We used Burkina Faso as the archetype, implementing this country’s demography and previous vaccination history. The model was coded and run in R using the package deSolve to perform the numerical integration of the differential equations using a time step of 1 day. Due to the stochastic nature of the seasonal forcing in the model, we performed 200 simulation runs. For each simulation, we ran the model for 100 years burn-in period. Results were recorded for 50 years starting from the year 2000 as the first year following the burn-in period. The average and range of the results from the 200 simulation runs are presented here.

### Vaccination

Vaccination is implemented in two different ways in the model according to the strategy modelled. For mass campaigns, we assume that these are discrete events taking place at one point in time. At that point in time, the targeted population is transferred from the unvaccinated states to the mirror vaccinated states. Ill people are not vaccinated. EPI is implemented continuously in the model as individuals reach the target age of 12 months (although the actual age of vaccination may be between 9 and 18 months we used 12 months as a simplification).

The absence of any observed menA cases in the African meningitis belt following vaccination with MenAfriVac suggests that duration of vaccine-induced protection is not short-lived. In the base case we assume that MenAfriVac and MMCV offer an average of 10 years protection for all ages. Different assumptions were investigated in a sensitivity analysis.

### Vaccination strategies modelled

We implemented MenAfriVac from 2010 as per the actual rollout [11]. We assumed that both campaigns with MenAfriVac, one in 2010 targeting 1–29 year olds and the catch-up campaign in 2016 of 1–6 year olds, achieved 100% coverage [11]. We further assumed 90% vaccination coverage for EPI which began in 2016 alongside the catch-up campaign. The same assumptions regarding the coverage were made for MMCV introduction in 2025 as well. We considered 3 different age targets for MMCV initial campaigns: 1–29 year olds; 1–19 year olds and 1–14 year olds. EPI vaccination at 12 months with MMCV was implemented at the same time as the campaigns. We also considered EPI only with no campaign. It was assumed that MenAfriVac would cease to be administered immediately after the rollout of MMCV. As the rollout of MMCVs is assumed to commence in 2025, the projected impact of MMCV implementation is evaluated over a 25-year period spanning 2025–2050. Beyond assessing the number of cases averted, we included the Number Needed to Vaccinate (NNV) as an additional metric to evaluate the relative efficiency of different vaccination strategies. In this case, the NNV is defined as the total number of doses administered divided by the total number of cases prevented under each vaccination strategy over the time period under consideration.

### Sensitivity analysis

We performed sensitivity analyses on several key parameters including: the duration of vaccine protection (average of 5, 10, 15 years or age-specific); vaccine effectiveness against carriage (VEC, 90% or 60%); relative transmission and invasion of menCWYX compared to menA. Age-specific protection assumes a shorter duration of protection for children under 5 years-olds than for those 5 years-old [12,13] and over and is intermediate between the 10 and 15 years duration.

## Results

**Fig 3:**
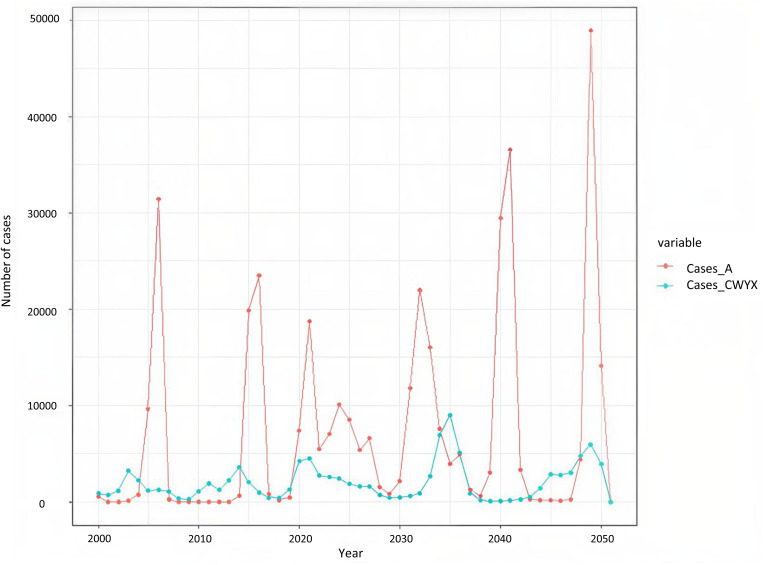
A typical run of the model with no vaccination.

In the absence of vaccination, the model was able to capture the unique epidemiology of meningococcal disease in the African meningitis belt with irregular epidemics of varying sizes. Fig 3 shows a typical model run showing epidemics caused by either menA or any of the serogroups C, W, Y and X at irregular intervals. This is not intended to faithfully reproduce past epidemics, rather simulate the typical patterns observed in high-burden countries.

### Numerical simulations for the base case (10 years duration of protection, 90% VE against carriage)

**Fig 4:**
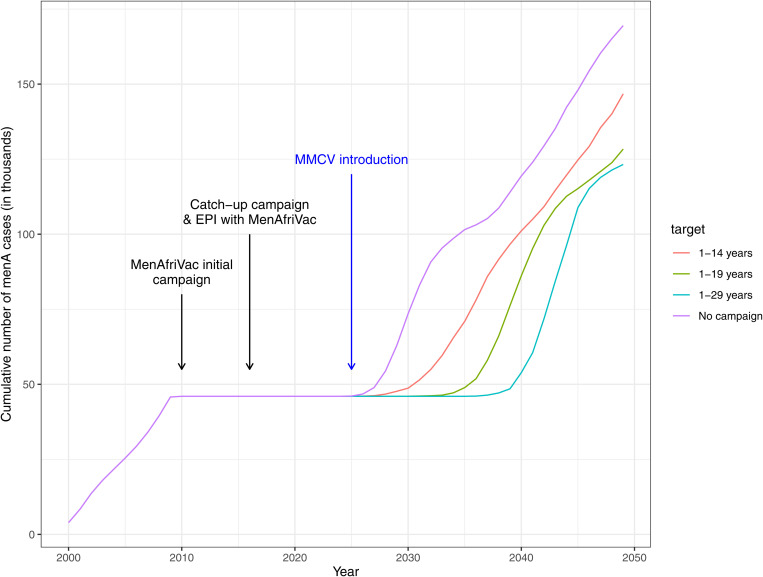
Cumulative number of menA cases during 2000-2050. The coloured lines represent the different scenarios simulated for MMCV introduction. Introduction of MMCV into EPI is common in all the strategies but the target of the introductory campaign differs. Average of 200 simulation runs.

**Fig 5:**
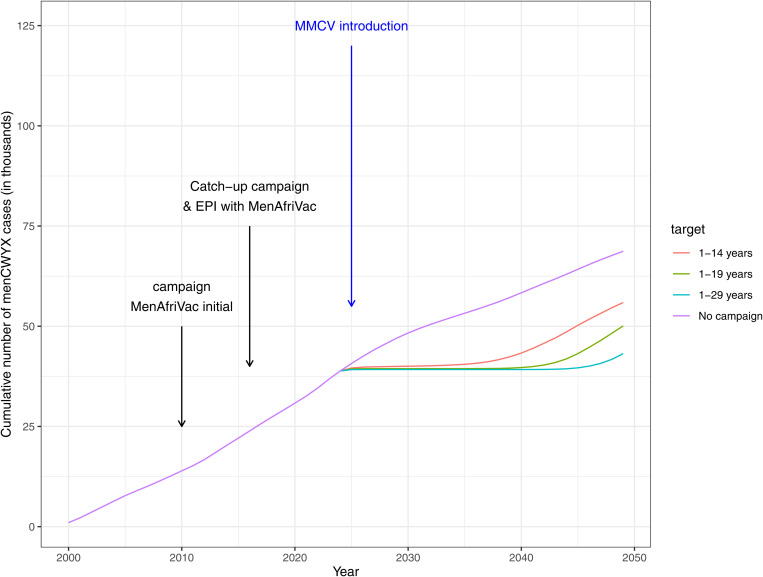
Cumulative number of menCWYX cases during 2000-2050. The coloured lines represent the different scenarios simulated for MMCV introduction. Introduction of MMCV into EPI is common in all the strategies but the target of the introductory campaign differs. Average of 200 simulation runs.

**Fig 6:**
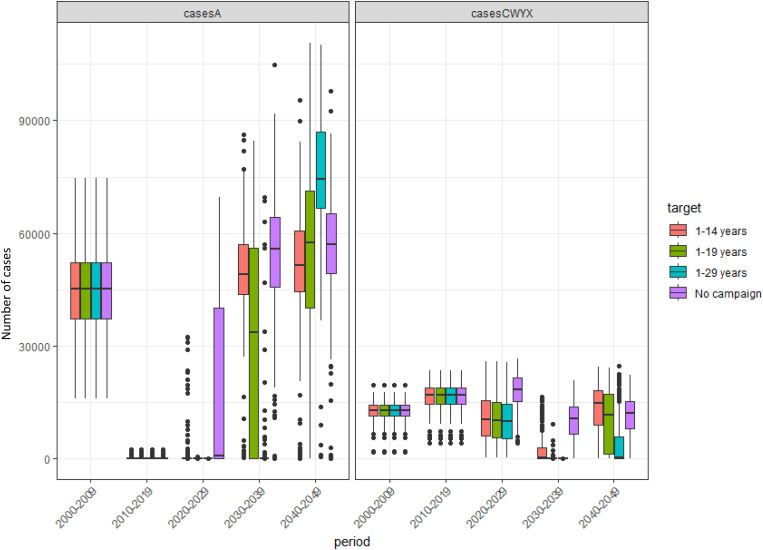
Predicted number of cases for different immunisation strategies. Box plot to show the median, interquartile range, and full range of the predicted number of cases by decade for different immunisation strategies with MMCV in the 40 years following MenAfriVac introduction from 200 model simulations. EPI at 12 months is common in all strategies.

Following the introduction of MenAfriVac in 2010, the model suggests a long honeymoon period with very few cases of group A. This is extended by the introduction of the multivalent meningococcal conjugate vaccine, which was rolled out through a mass campaign in 2025 and simultaneously replaced MenAfriVac in the EPI schedule, effectively providing a boost for group A and then we start to see disease re-emerging in 2030–2040. This emergence is more rapid if the target age range for MMCV is narrower. So, for the 1–14 years target we start to see disease incidence rising earlier than when targeting 1–29 years (Fig 4). For group CWYX the impact is shown mostly in 2030 onwards (Fig 5). There is no difference in earlier years because vaccine is introduced in 2025. The impact is greater if a larger population is targeted for the mass campaign. The ‘No campaign’ scenario in Figs 4 and 5 refers to MMCV being introduced only in EPI; this has a less of an impact than a scenario with a campaign, however, there is some benefit compared to no MMCV vaccination. Boxplots showing the full range of results by decade can be seen in Fig 6.

In terms of NNV over a 25 year period, again this follows the pattern of results suggesting that the 1–29 year-olds target is the best (Table 3). However, the NNV for the 1–29 y/o campaigns is much closer to the NNV for the 1–19 y/o campaigns than it is for the 1–14 year-olds campaign.

**Table 3:** Numerical results across the different assumptions.

MMCV target	Vaccine doses	MenA cases averted	MenCWYX cases averted	Total cases averted	Duration of protection	NNV
1-29 y/o	38688453	48349	58079	106428	10 years	364
1-19 y/o	34563000	38827	51002	89829	10 years	385
1-14 y/o	31287943	20810	45716	66526	10 years	470

Number of cases averted and NNV across the different assumptions regarding the target group for MMCV campaign, assessed over a 25 year period.

**Fig 7:**
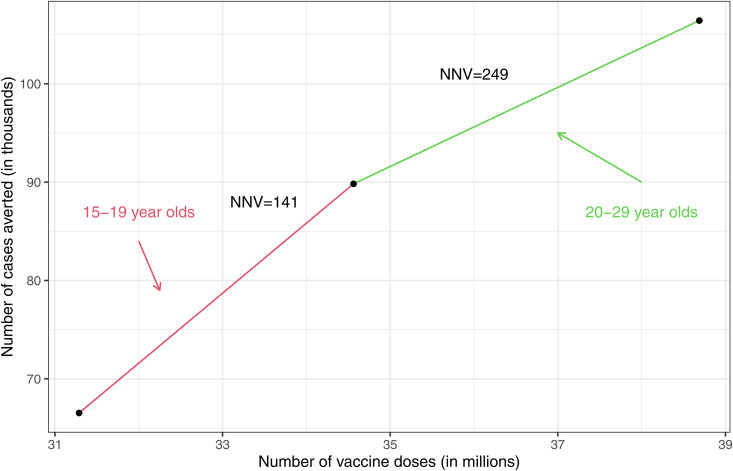
Predicted number of cases averted. Plot showing the number of cases averted over a 25 year time period predicted by the model against the number of required vaccine doses increasing the target age group incrementally from 1-14 years to include 15-19 year olds and then 20-29 year olds. EPI is common in all scenarios.

To better examine the efficiency of each strategy, we estimated the benefit of increasing the target group incrementally from 1–14 y/o to include 15–19 y/o and then 20–29 y/o. As shown in Fig 7, including 15–19 year-olds to the target population results in 23,303 cases more cases being averted than targeting 1–14 year olds alone with an additional number of vaccine doses of 3.27 million. A further 4.12 million vaccine doses needed to immunise the 20–29 year-olds is estimated to prevent 16,599 cases during the 25 year period between 2025 and 2050.

### Sensitivity analyses

**Fig 8:**
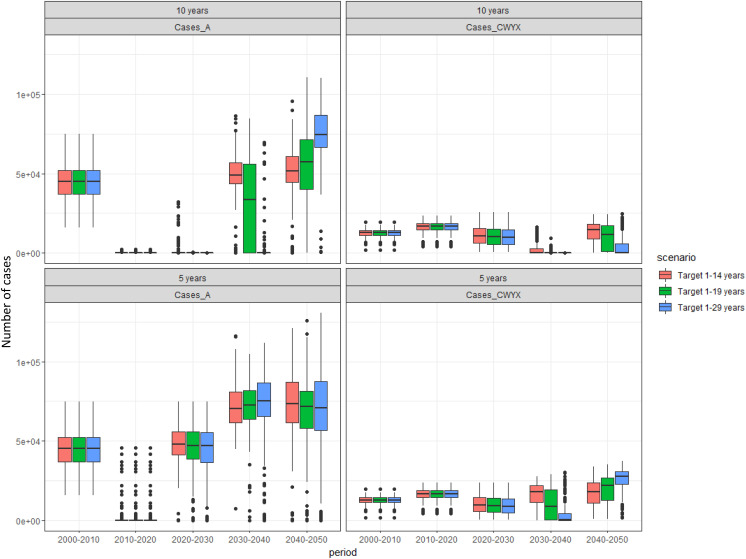
Predicted number of cases for 5 and 10 years assumed duration of protection. Box plots to show the median, interquartile range, and full range of the predicted number of cases by decade for different immunisation strategies with MMCV in the 40 years following MenAfriVac introduction from 200 model simulations across different assumptions regarding the duration of protection. EPI is common in all strategies.

**Fig 9:**
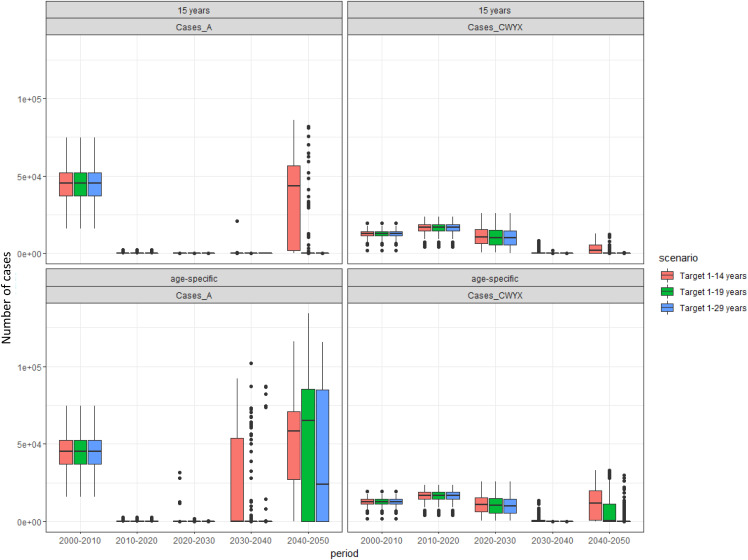
Predicted number of cases for age-specific and 15 years assumed duration of protection. Box plots to show the median, interquartile range, and full range of the predicted number of cases by decade for different immunisation strategies with MMCV in the 40 years following MenAfriVac introduction from 200 model simulations across different assumptions regarding the duration of protection. EPI is common in all strategies.

Sensitivity analysis on the duration of protection showed that shorter duration of protection results in a lower impact on disease burden, while a longer assumed duration of protection extends the period with very low disease incidence with both MenA and MenCWYX (Figs 8 and 9). Disease is tending towards elimination assuming an average duration of protection of 15 years. The results of this analysis are summarised in Tables 4 and 5 showing the predicted year of resurgence and the calculated NNV across all different assumptions, respectively. The larger the population is immunised, the longer the protection lasts. Similarly to the base case scenario, the NNV for the 1–29 y/o is much closer to the NNV for the 1–19 y/o than it is for the 1–14 y/o.

**Table 4:** Predicted year of resurgence.

Duration of protection	1-14 y/o		1-19 y/o		1-29 y/o	
	A	CWYX	A	CWYX	A	CWYX
10 years (base case)	2028	2034	2034	2040	2038	2044
5 years	2026	2030	2029	2034	2031	2037
Age-specific	2031	2037	2036	2043	2037	2047
15 years	2042	2038	2047	2049	n/a	n/a

Resurgence is defined as 400 cases for MenA and 100 cases for MenCWYX.

**Table 5:** Summary of results.

MMCV target	Vaccine doses	MenA cases averted	MenCWYX cases averted	Total cases averted	Duration of protection	NNV
1-29 y/o	38688453	25912	33267	59179	5 years	654
1-19 y/o	34563000	21747	31801	53548	5 years	645
1-14 y/o	31287943	16559	27758	44317	5 years	706
1-29 y/o	38688453	48349	58079	106428	10 years	364
1-19 y/o	34563000	38827	51002	89829	10 years	385
1-14 y/o	31287943	20810	45716	66526	10 years	470
1-29 y/o	38688453	60783	60299	121082	Age-specific	320
1-19 y/o	34563000	45623	55280	100903	Age-specific	343
1-14 y/o	31287943	31614	48793	80407	Age-specific	389
1-29 y/o	38688453	63437	63979	127416	15 years	304
1-19 y/o	34563000	55733	63051	118784	15 years	291
1-14 y/o	31287943	20093	58815	78908	15 years	397

Number of cases averted and NNV across the different assumptions regarding the target group for MMCV campaign and the duration of protection.

**Fig 10:**
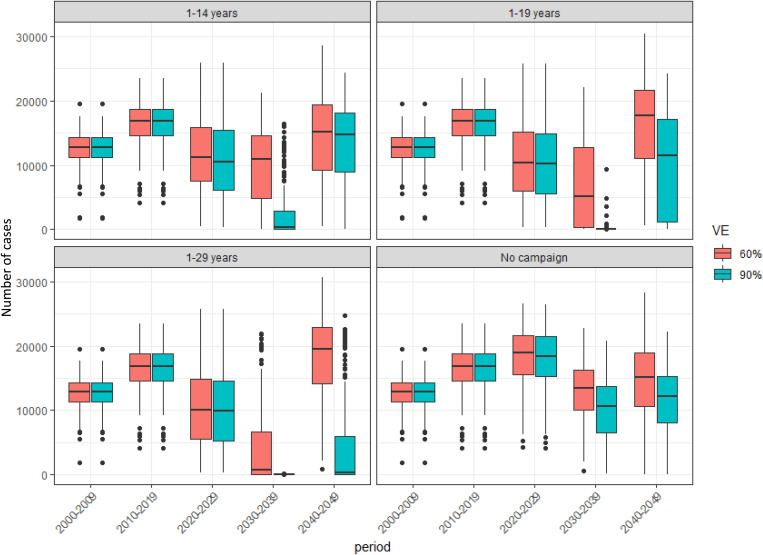
Predicted number of cases for different VEC assumptions. Box plots to show the median, interquartile range, and full range of the predicted number of menCWYX cases by decade for different VEC assumptions. Average assumed duration of protection is 10 years. From 200 simulation runs.

We examined the sensitivity of our results to assumptions on vaccine effectiveness against carriage (VEC). We ran our simulations keeping the VEC for MenAfriVac at 90% (for which there is empirical evidence [14]) and reduced the VEC for MMCV to 60%. A lower bound of 60% VEC was chosen to reflect conservative assumptions based on UK MenACWY observational data, where effectiveness against invasive disease and/or carriage acquisition has ranged between 60% and 90% depending on cohort and follow-up time. As expected, the results showed that 90% VEC leads to more cases being prevented than assuming 60% VEC with disease due to MenCWYX returning earlier if the VEC is 60% (Fig 10). The difference in disease burden estimated assuming 90% and 60% VEC for MMCVs is more profound when the target age group for the campaign is smaller. In terms of the NNV, the pattern is the same as assuming 90% VEC.

## Discussion

We adapted our previous model to include carriage and disease with MenCWYX in order to simulate the impact of MMCVs against disease due to MenACWYX. We estimated that a campaign targeting 1–29 year olds would result in the highest number of cases averted and the longest time to resurgence. In a situation where resources are constrained, we found that targeting 1–19 y/o is a much better choice than targeting 1–14 y/o because the differential between the 1–14 y/o and 1–19 y/o is greater than the differential between 1–19 y/o and 1–29 y/o. The duration of protection is a parameter which matters in terms of year to resurgence. The results are sensitive to the duration of protection assumed but the order of preference among the different scenarios remains. Vaccine efficacy against carriage of MenCWYX is a very influential parameter that we do not know the true value of, this should be investigated in empirical studies. Sensitivity analysis on this parameter showed that the ranking of the strategies does not change but there is much greater difference in the number of cases prevented at 90% VE than at 60% VE. We further looked at EPI-only vaccination where we did not have a preventive campaign. Using Burkina Faso as an example only, we showed that there is some value in EPI only vaccination; this could be a good option for lower risk countries without significant burden of disease in older children and adults.

Our work has several strengths and limitations. The model is an extension of our previous model that looked at the transmission of MenA only. As such, we feel we have a good understanding of the underlying system dynamics. There are limited data to inform the model, especially from low-risk countries, and we performed sensitivity analyses on key parameters to investigate parameter uncertainty. We have included a year to year variation in disease incidence, however, we have not accounted for the introduction of a new hypervirulent strain which may be associated with higher epidemic risk and overall burden of disease. Periods of very low carriage and disease incidence may lead to elimination. A different type of model (e.g., a stochastic transmission model) is needed to explore this and is the subject of further research. Our analysis assumes 90% EPI coverage and 100% campaign coverage among unvaccinated individuals to reflect best-case delivery scenarios. These values align with global policy targets, such as those set by Gavi and IA2030, and were chosen to allow for consistent comparison across intervention strategies. However, we recognise that in practice, coverage is often lower and more variable. These assumptions may overestimate vaccine impact in real-world settings, and this should be considered when interpreting the results. A more detailed exploration of coverage variability is important but lies beyond the scope of this study.

Following the success of MenAfriVac, countries will now need to consider how to best sustain protection against group A and also protect the population from other meningococcal groups causing epidemics in the African meningitis belt using multivalent conjugate vaccines. Findings from mathematical models such as this can lend further support to decision makers on which is the most efficient introduction of MMCVs in the belt, as well as help identify critical evidence gaps and priority research for their decisions. The recommendations from SAGE are that all countries in the African meningitis belt introduce the novel MMCV into their routine immunisation programs. In high-risk countries, the introduction of MMCV into EPI should be accompanied by a catch-up campaign targeting 1–19 year olds [15].

## Supporting information

S1 Appendix Model differential equations. (DOCX)
