# Supplementary material for: Modelling of strategies for the introduction and routine use of multivalent meningococcal conjugate vaccines (MMCVs) in the African meningitis belt
Source: PLoS One. 2025 Aug 29;20(8):e0330627. doi: 10.1371/journal.pone.0330627 (PMC12396689; doi:10.1371/journal.pone.0330627)
Supplement: S1 Appendix — (DOCX) [file pone.0330627.s001.docx]

Supplementary information

Equations

When i=1 (first age group)

Unvaccinated individuals

$$\frac{dS\left( 1 \right)}{dt}=B+\varphi R_{A}\left( 1 \right)+\varphi R_{CWYX}\left( 1 \right)+\varphi R\left( 1 \right)-\lambda_{A}\left( 1 \right)S\left( 1 \right)-\lambda_{CWYX}\left( 1 \right)S\left( 1 \right)-\left( K\left( 1 \right)+ mu\left( 1 \right) \right)S\left( 1 \right)+ w\left( 1 \right)PS\left( 1 \right)+w\left( 1 \right)MS\left( 1 \right)$$

$$\frac{d{C1}_{A}\left( 1 \right)}{dt}=-\left( a_{A}\left( 1 \right)+\alpha_{A}+K\left( 1 \right)+mu\left( 1 \right) \right){C1}_{A}\left( 1 \right)+\lambda_{A}\left( 1 \right)S\left( 1 \right)+w\left( 1 \right)P{C1}_{A}\left( 1 \right)+ w\left( 1 \right)M{C1}_{A}\left( 1 \right)$$

$$\frac{d{C1}_{CWYX}\left( 1 \right)}{dt}=-\left( a_{CWYX}\left( 1 \right)+\alpha_{CWYX}+K\left( 1 \right)+mu\left( 1 \right) \right){C1}_{CWYX}\left( 1 \right)+\lambda_{CWYX}\left( 1 \right)S\left( 1 \right)+w\left( 1 \right)P{C1}_{CWYX}\left( 1 \right)+\boldsymbol{w}\left( 1 \right)\boldsymbol{M}{C1}_{CWYX}\left( 1 \right)$$

$$\frac{d{I1}_{A}\left( 1 \right)}{dt}=a_{A}\left( 1 \right){C1}_{A}\left( 1 \right)-\left( \rho+K\left( 1 \right)+mu\left( 1 \right) \right){I1}_{A}\left( 1 \right)$$

$$\frac{d{I1}_{CWYX}\left( 1 \right)}{dt}=a_{CWYX}\left( 1 \right){C1}_{CWYX}\left( 1 \right)-\left( \rho+K\left( 1 \right)+mu\left( 1 \right) \right){I1}_{CWYX}\left( 1 \right)$$

$$\frac{dR_{A}\left( 1 \right)}{dt}= \rho{I1}_{A}\left( 1 \right)+\alpha_{A}{C1}_{A}\left( 1 \right)-\left( \varphi+K\left( 1 \right)+mu\left( 1 \right) \right)R_{A}\left( 1 \right)+w\left( 1 \right)PR_{A}\left( 1 \right)+w\left( 1 \right)MR_{A}\left( 1 \right)$$

$$\frac{dR_{CWYX}\left( 1 \right)}{dt}= \rho{I1}_{CWYX}\left( 1 \right)+\alpha_{CWYX}{C1}_{CWYX}\left( 1 \right)-\left( \varphi+K\left( 1 \right)+mu\left( 1 \right) \right)R_{CWYX}\left( 1 \right)+w\left( 1 \right)PR_{CWYX}\left( 1 \right)+w\left( 1 \right)MR_{CWYX}\left( 1 \right)$$

$$\frac{d{C2}_{A}\left( 1 \right)}{dt}=-\left( a_{A}\left( 1 \right)+\alpha_{A}+K\left( 1 \right)+mu\left( 1 \right) \right){C2}_{A}\left( 1 \right)+\lambda_{A}\left( 1 \right)R_{CWYX}\left( 1 \right)+w\left( 1 \right)P{C2}_{A}\left( 1 \right)+w\left( 1 \right)M{C2}_{A}\left( 1 \right)$$

$$\frac{d{C2}_{CWYX}\left( 1 \right)}{dt}=-\left( a_{CWYX}\left( 1 \right)+\alpha_{CWYX}+K\left( 1 \right)+mu\left( 1 \right) \right){C2}_{CWYX}\left( 1 \right)+\lambda_{CWYX}\left( 1 \right)R_{A}\left( 1 \right)+w\left( 1 \right)P{C2}_{CWYX}\left( 1 \right)+w\left( 1 \right)M{C2}_{CWYX}\left( 1 \right)$$

$$\frac{d{I2}_{A}\left( 1 \right)}{dt}=a_{A}\left( 1 \right){C2}_{A}\left( 1 \right)-\left( \rho+K\left( 1 \right)+mu\left( 1 \right) \right){I2}_{A}\left( 1 \right)$$

$$\frac{d{I2}_{CWYX}\left( 1 \right)}{dt}=a_{CWYX}\left( 1 \right){C2}_{CWYX}\left( 1 \right)-\left( \rho+K\left( 1 \right)+mu\left( 1 \right) \right){I2}_{CWYX}\left( 1 \right)$$

$$\frac{dR\left( 1 \right)}{dt}= \rho{I2}_{A}\left( 1 \right)+\rho{I2}_{CWYX}\left( 1 \right)+\alpha_{A}{C2}_{A}\left( 1 \right)+\alpha_{CWYX}{C2}_{CWYX}\left( 1 \right)-\left( \varphi+K\left( 1 \right)+mu\left( 1 \right) \right)R\left( 1 \right)+w\left( 1 \right)PR\left( 1 \right)+w\left( 1 \right)MR\left( 1 \right)$$

For i = 2,…100 (annual age cohorts)

Unvaccinated individuals

$$\frac{dS\left( i \right)}{dt}=\varphi R_{A}\left( i \right)+\varphi R_{CWYX}\left( i \right)+\varphi R\left( i \right)-\lambda_{A}\left( i \right)S\left( i \right)-\lambda_{CWYX}\left( i \right)S\left( i \right)-\left( K\left( i \right)+ mu\left( i \right) \right)S\left( i \right)+ w\left( i \right)PS\left( i \right)+w\left( i \right)MS\left( i \right)+\left( 1-\gamma_{A}\left( i \right)-\gamma_{CWYX}\left( i \right) \right)K(i-1)S\left( i-1 \right)$$

$$\frac{d{C1}_{A}\left( i \right)}{dt}=-\left( a_{A}\left( i \right)+\alpha_{A}+K\left( i \right)+mu\left( i \right) \right){C1}_{A}\left( i \right)+\lambda_{A}\left( i \right)S\left( i \right)+w\left( i \right)P{C1}_{A}\left( i \right)+ w\left( i \right)M{C1}_{A}\left( i \right)-\left( 1-\gamma_{A}\left( i \right)-\gamma_{CWYX}\left( i \right) \right)K\left( i-1 \right){C1}_{A}\left( i-1 \right)$$

$$\frac{d{C1}_{CWYX}\left( i \right)}{dt}=-\left( a_{CWYX}\left( i \right)+\alpha_{CWYX}+K\left( i \right)+mu\left( i \right) \right){C1}_{CWYX}\left( i \right)+\lambda_{CWYX}\left( i \right)S\left( i \right)+w\left( i \right)P{C1}_{CWYX}\left( i \right)+\boldsymbol{w}\left( i \right)\boldsymbol{M}{C1}_{CWYX}\left( i \right)-\left( 1-\gamma_{A}\left( i \right)-\gamma_{CWYX}\left( i \right) \right){K\left( i-1 \right)C1}_{CWYX}\left( i-1 \right)$$

$\frac{d{I1}_{A}\left( i \right)}{dt}=a_{A}\left( i \right){C1}_{A}\left( i \right)-\left( \rho+K\left( i \right)+mu\left( i \right) \right){I1}_{A}\left( i \right)$+ $K\left( i-1 \right){I1}_{A}\left( i-1 \right)$

$$\frac{d{I1}_{CWYX}\left( i \right)}{dt}=a_{CWYX}\left( i \right){C1}_{CWYX}\left( i \right)-\left( \rho+K\left( i \right)+mu\left( i \right) \right){I1}_{CWYX}\left( i \right)+ K\left( i-1 \right){I1}_{CWYX}\left( i-1 \right)$$

$$\frac{dR_{A}\left( i \right)}{dt}= \rho{I1}_{A}\left( i \right)+\alpha_{A}{C1}_{A}\left( i \right)-\left( \varphi+K\left( i \right)+mu\left( i \right) \right)R_{A}\left( i \right)+w\left( i \right)PR_{A}\left( i \right)+w\left( i \right)MR_{A}\left( i \right)-\left( 1-\gamma_{A}\left( i \right)-\gamma_{CWYX}\left( i \right) \right)K(i-1)R_{A}\left( i-1 \right)$$

$$\frac{dR_{CWYX}\left( i \right)}{dt}= \rho{I1}_{CWYX}\left( i \right)+\alpha_{CWYX}{C1}_{CWYX}\left( i \right)-\left( \varphi+K\left( i \right)+mu\left( i \right) \right)R_{CWYX}\left( i \right)+w\left( i \right)PR_{CWYX}\left( i \right)+w\left( i \right)MR_{CWYX}\left( i \right)-\left( 1-\gamma_{A}\left( i \right)-\gamma_{CWYX}\left( i \right) \right)K(i-1)R_{CWYX}\left( i-1 \right)$$

$$\frac{d{C2}_{A}\left( i \right)}{dt}=-\left( a_{A}\left( i \right)+\alpha_{A}+K\left( i \right)+mu\left( i \right) \right){C2}_{A}\left( i \right)+\lambda_{A}\left( i \right)R_{CWYX}\left( i \right)+w\left( i \right)P{C2}_{A}\left( i \right)+w\left( i \right)M{C2}_{A}\left( i \right)-\left( 1-\gamma_{A}\left( i \right)-\gamma_{CWYX}\left( i \right) \right)K(i-1){C2}_{A}\left( i-1 \right)$$

$$\frac{d{C2}_{CWYX}\left( i \right)}{dt}=-\left( a_{CWYX}\left( i \right)+\alpha_{CWYX}+K\left( i \right)+mu\left( i \right) \right){C2}_{CWYX}\left( i \right)+\lambda_{CWYX}\left( i \right)R_{A}\left( i \right)+w\left( i \right)P{C2}_{CWYX}\left( i \right)+w\left( i \right)M{C2}_{CWYX}\left( i \right)-\left( 1-\gamma_{A}\left( i \right)-\gamma_{CWYX}\left( i \right) \right)K(i-1){C2}_{CWYX}\left( i-1 \right)$$

$$\frac{d{I2}_{A}\left( i \right)}{dt}=a_{A}\left( i \right){C2}_{A}\left( i \right)-\left( \rho+K\left( i \right)+mu\left( i \right) \right){I2}_{A}\left( i \right)+K(i-1){I2}_{A}\left( i-1 \right)$$

$$\frac{d{I2}_{CWYX}\left( i \right)}{dt}=a_{CWYX}\left( i \right){C2}_{CWYX}\left( i \right)-\left( \rho+K\left( i \right)+mu\left( i \right) \right){I2}_{CWYX}\left( i \right)+K(i-1){I2}_{CWYX}\left( i-1 \right)$$

$$\frac{dR\left( i \right)}{dt}= \rho{I2}_{A}\left( i \right)+\rho{I2}_{CWYX}\left( i \right)+\alpha_{A}{C2}_{A}\left( i \right)+\alpha_{CWYX}{C2}_{CWYX}\left( i \right)-\left( \varphi+K\left( i \right)+mu\left( i \right) \right)R\left( i \right)+w\left( i \right)PR\left( i \right)+w\left( i \right)MR\left( i \right)-\left( 1-\gamma_{A}\left( i \right)-\gamma_{CWYX}\left( i \right) \right)K(i-1)R\left( i-1 \right)$$

Vaccinated with MenAfriVac

$$\frac{dMS\left( i \right)}{dt}=\varphi{MR}_{A}\left( i \right)+\varphi MR_{CWYX}\left( i \right)+\varphi MR\left( i \right)-\left( 1-\delta_{A} \right)\lambda_{A}\left( i \right)MS\left( i \right)-\lambda_{CWYX}\left( i \right)MS\left( i \right)-\left( K\left( i \right)+ mu\left( i \right) \right)MS\left( i \right)-w\left( i \right)MS\left( i \right)+\gamma_{A}\left( i \right)K(i-1)S(i-1) +K(i-1)MS\left( i-1 \right)$$

$$\frac{dM{C1}_{A}\left( i \right)}{dt}=-\left( \left( 1-\xi_{A} \right)a_{A}\left( i \right)+\alpha_{A}+K\left( i \right)+mu\left( i \right) \right){MC1}_{A}\left( i \right)+\left( 1-\delta_{A} \right)\lambda_{A}\left( i \right)MS\left( i \right)- w\left( i \right)M{C1}_{A}\left( i \right)+\gamma_{A}\left( i \right)K\left( i-1 \right){C1}_{A}\left( i-1 \right)+K(i-1)M{C1}_{A}\left( i-1 \right)$$

$$\frac{dM{C1}_{CWYX}\left( i \right)}{dt}=-\left( a_{CWYX}\left( i \right)+\alpha_{CWYX}+K\left( i \right)+mu\left( i \right) \right)M{C1}_{CWYX}\left( i \right)+\lambda_{CWYX}\left( i \right)MS\left( i \right)-\boldsymbol{w}\left( i \right)\boldsymbol{M}{C1}_{CWYX}\left( i \right)+\gamma_{A}\left( i \right){K(i-1)C1}_{CWYX}\left( i-1 \right) +K(i-1)M{C1}_{CWYX}\left( i-1 \right)$$

$$\frac{d{MI1}_{A}\left( i \right)}{dt}=\left( 1-\xi_{A} \right)a_{A}\left( i \right)M{C1}_{A}\left( i \right)-\left( \rho+K\left( i \right)+mu\left( i \right) \right){MI1}_{A}\left( i \right)+K(i-1)MI_{A}\left( i-1 \right)$$

$$\frac{d{MI1}_{CWYX}\left( i \right)}{dt}=a_{CWYX}\left( i \right)M{C1}_{CWYX}\left( i \right)-\left( \rho+K\left( i \right)+mu\left( i \right) \right){MI1}_{CWYX}\left( i \right)+K(i-1)M{I1}_{CWYX}\left( i-1 \right)$$

$$\frac{dMR_{A}\left( i \right)}{dt}= \rho M{I1}_{A}\left( i \right)+\alpha_{A}M{C1}_{A}\left( i \right)-\left( \varphi+K\left( i \right)+mu\left( i \right) \right){MR}_{A}\left( i \right)-w\left( i \right)MR_{A}\left( i \right)+\gamma_{A}\left( i \right){K(i-1)R}_{A}\left( i-1 \right)+K(i-1)MR_{A}\left( i-1 \right)$$

$$\frac{dMR_{CWYX}\left( i \right)}{dt}= \rho M{I1}_{CWYX}\left( i \right)+\alpha_{CWYX}M{C1}_{CWYX}\left( i \right)-\left( \varphi+K\left( i \right)-K\left( i-1 \right)+mu\left( i \right) \right)MR_{CWYX}\left( i \right)-w\left( i \right)MR_{CWYX}\left( i \right)+\gamma_{A}\left( i \right){K(i-1)R}_{CWYX}\left( i-1 \right) +K(i-1)MR_{CWYX}\left( i-1 \right)$$

$$\frac{dM{C2}_{A}\left( i \right)}{dt}=-\left( \left( 1-\xi_{A} \right)a_{A}\left( i \right)+\alpha_{A}+K\left( i \right)+mu\left( i \right) \right){MC2}_{A}\left( i \right)+\left( 1-\delta_{A} \right)\lambda_{A}\left( i \right)MR_{CWYX}\left( i \right)-w\left( i \right)M{C2}_{A}\left( i \right)+\gamma_{A}\left( i \right){K(i-1)C2}_{A}\left( i-1 \right)+K(i-1)M{C2}_{A}\left( i-1 \right)$$

$$\frac{d{MC2}_{CWYX}\left( i \right)}{dt}=-\left( a_{CWYX}\left( i \right)+\alpha_{CWYX}+K\left( i \right)+mu\left( i \right) \right)M{C2}_{CWYX}\left( i \right)+\lambda_{CWYX}\left( i \right)MR_{A}\left( i \right)-w\left( i \right)M{C2}_{CWYX}\left( i \right)+\gamma_{A}\left( i \right){K(i-1)C2}_{CWYX}\left( i-1 \right)+K(i-1)M{C2}_{CWYX}\left( i-1 \right)$$

$$\frac{dM{I2}_{A}\left( i \right)}{dt}=\left( 1-\xi_{A} \right)a_{A}\left( i \right)M{C2}_{A}\left( i \right)-\left( \rho+K\left( i \right)+mu\left( i \right) \right){MI2}_{A}\left( i \right)+K(i-1)M{I2}_{A}\left( i-1 \right)$$

$$\frac{d{MI2}_{CWYX}\left( i \right)}{dt}=a_{CWYX}\left( i \right)M{C2}_{CWYX}\left( i \right)-\left( \rho+K\left( 1 \right)+mu\left( i \right) \right){MI2}_{CWYX}\left( i \right)+K(i-1)M{I2}_{CWYX}\left( i-1 \right)$$

$$\frac{dMR\left( i \right)}{dt}= \rho{MI2}_{A}\left( i \right)+\rho M{I2}_{CWYX}\left( i \right)+\alpha_{A}M{C2}_{A}\left( i \right)+\alpha_{CWYX}M{C2}_{CWYX}\left( i \right)-\left( \varphi+K\left( i \right)+mu\left( i \right) \right)MR\left( i \right)-w\left( i \right)MR\left( i \right)+\gamma_{A}\left( i \right)K(i-1)R\left( i \right)+K(i-1)MR\left( i-1 \right)$$

Vaccinated with Pentavalent

$$\frac{dPS\left( i \right)}{dt}=\varphi{PR}_{A}\left( i \right)+\varphi PR_{CWYX}\left( i \right)+\varphi PR\left( i \right)-\left( 1-\delta_{A} \right)\lambda_{A}\left( i \right)PS\left( i \right)-\left( 1-\delta_{CWYX} \right)\lambda_{CWYX}\left( i \right)PS\left( i \right)-\left( K\left( i \right)+ mu\left( i \right) \right)PS\left( i \right)-w\left( i \right)PS\left( i \right)+\gamma_{CWYX}\left( i \right)K\left( i-1 \right)S(i-1)+K(i-1)PS\left( i-1 \right)$$

$$\frac{dP{C1}_{A}\left( i \right)}{dt}=-\left( \left( 1-\xi_{A} \right)a_{A}\left( i \right)+\alpha_{A}+K\left( i \right)+mu\left( i \right) \right){PC1}_{A}\left( i \right)+\left( 1-\delta_{A} \right)\lambda_{A}\left( i \right)PS\left( i \right)- w\left( i \right)P{C1}_{A}\left( i \right)+\gamma_{CWYX}\left( i \right)K(i-1){C1}_{A}\left( i-1 \right) +K(i-1){PC1}_{A}\left( i-1 \right)$$

$$\frac{dP{C1}_{CWYX}\left( i \right)}{dt}=-\left( \left( 1-\xi_{CWYX} \right)a_{CWYX}\left( i \right)+\alpha_{CWYX}+K\left( i \right)+mu\left( i \right) \right)P{C1}_{CWYX}\left( i \right)+\left( 1-\delta_{CWYX} \right)\lambda_{CWYX}\left( i \right)PS\left( i \right)-\boldsymbol{w}\left( i \right)\boldsymbol{M}P\left( i \right)+\gamma_{CWYX}\left( i \right)K(i-1){C1}_{CWYX}\left( i-1 \right)+K(i-1){PC1}_{CWYX}\left( i-1 \right)$$

$$\frac{d{PI1}_{A}\left( i \right)}{dt}=\left( 1-\xi_{A} \right)a_{A}\left( i \right)P{C1}_{A}\left( i \right)-\left( \rho+K\left( i \right)+mu\left( i \right) \right){PI1}_{A}\left( i \right)+K(i-1){PI1}_{A}\left( i-1 \right)$$

$$\frac{d{PI1}_{CWYX}\left( i \right)}{dt}=\left( 1-\xi_{CWYX} \right)a_{CWYX}\left( i \right)P{C1}_{CWYX}\left( i \right)-\left( \rho+K\left( i \right)+mu\left( i \right) \right){PI1}_{CWYX}\left( i \right)+K(i-1){PC1}_{CWYX}\left( i-1 \right)$$

$$\frac{dPR_{A}\left( i \right)}{dt}= \rho P{I1}_{A}\left( i \right)+\alpha_{A}P{C1}_{A}\left( i \right)-\left( \varphi+K\left( i \right)+mu\left( i \right) \right){PR}_{A}\left( i \right)-w\left( i \right)PR_{A}\left( i \right)+\gamma_{CWYX}\left( i \right){K(i-1)R}_{A}\left( i-1 \right)+K(i-1){PR}_{A}\left( i-1 \right)$$

$$\frac{dPR_{CWYX}\left( i \right)}{dt}= \rho P{I1}_{CWYX}\left( i \right)+\alpha_{CWYX}P{C1}_{CWYX}\left( i \right)-\left( \varphi+K\left( i \right)+mu\left( i \right) \right)PR_{CWYX}\left( i \right)-w\left( i \right)PR_{CWYX}\left( i \right)+\gamma_{CWYX}\left( i \right){K(i-1)R}_{CWYX}\left( i-1 \right)+K(i-1){PR}_{CWYX}\left( i-1 \right)$$

$$\frac{dP{C2}_{A}\left( i \right)}{dt}=-\left( \left( 1-\xi_{A} \right)a_{A}\left( i \right)+\alpha_{A}+K\left( i \right)+mu\left( i \right) \right){PC2}_{A}\left( i \right)+\left( 1-\delta_{A} \right)\lambda_{A}\left( i \right)PR_{CWYX}\left( i \right)-w\left( i \right)P{C2}_{A}\left( i \right)+\gamma_{CWYX}\left( i \right){K(i-1)C2}_{A}\left( i-1 \right)+K(i-1){PC2}_{A}\left( i-1 \right)$$

$$\frac{d{PC2}_{CWYX}\left( i \right)}{dt}=-\left( {\left( 1-\xi_{CWYX} \right)a}_{CWYX}\left( i \right)+\alpha_{CWYX}+K\left( i \right)+mu\left( i \right) \right)P{C2}_{CWYX}\left( i \right)+{\left( 1-\delta_{CWYX} \right)\lambda}_{CWYX}\left( i \right)PR_{A}\left( i \right)-w\left( i \right)P{C2}_{CWYX}\left( i \right)+\gamma_{CWYX}\left( i \right)K(i-1){C2}_{CWYX}\left( i-1 \right)+K(i-1){PC2}_{CWYX}\left( i-1 \right)$$

$$\frac{dP{I2}_{A}\left( i \right)}{dt}=\left( 1-\xi_{A} \right)a_{A}\left( i \right)P{C2}_{A}\left( i \right)-\left( \rho+K\left( i \right)+mu\left( i \right) \right){PI2}_{A}\left( i \right)+K(i-1){PI2}_{A}\left( i-1 \right)$$

$$\frac{d{PI2}_{CWYX}\left( i \right)}{dt}=\left( 1-\xi_{CWYX} \right)a_{CWYX}\left( i \right)P{C2}_{CWYX}\left( i \right)-\left( \rho+K\left( 1 \right)+mu\left( i \right) \right){PI2}_{CWYX}\left( i \right)+K(i-1){PI2}_{CWYX}\left( i-1 \right)$$

$$\frac{dPR\left( i \right)}{dt}= \rho{PI2}_{A}\left( i \right)+\rho P{I2}_{CWYX}\left( i \right)+\alpha_{A}P{C2}_{A}\left( i \right)+\alpha_{CWYX}P{C2}_{CWYX}\left( i \right)-\left( \varphi+K\left( i \right)+mu\left( i \right) \right)PR\left( i \right)-w\left( i \right)PR\left( i \right)+\gamma_{CWYX}\left( i \right)K(i-1)R\left( i-1 \right)+K(i-1)PR\left( i-1 \right)$$

People are born into the susceptible compartment at a rate B. The ageing rate K is as follows:

$$K=\left\{ \begin{aligned} \frac{1}{365}{day}^{-1} \\ 0 ,when i=99 \end{aligned} \right., when i=1,2,\ldots,99$$

Vaccination coverage γ is:

$$\gamma= \left\{ \begin{aligned} 0.9, for i=2 \\ 0, else \end{aligned} \right.$$
